# Trastuzumab, leuprorelin, letrozole, and palbociclib as first-line therapy in HER2-positive and hormone receptor-positive metastatic breast cancer: A case report

**DOI:** 10.1097/MD.0000000000033975

**Published:** 2023-06-16

**Authors:** Maoben Sun, Liangzhen Cai, Min Chen

**Affiliations:** a Department of Oncology, Binhaiwan Central Hospital, Dongguan, Guangdong, China.

**Keywords:** case report, letrozole, leuprorelin, metastatic breast cancer, palbociclib, trastuzumab

## Abstract

Cyclin-dependent kinase 4/6 inhibitors are promising candidates for patients with human epidermal growth factor receptor 2 (HER2)-positive breast cancer. However, current international guidelines recommend endocrine therapy alone or with HER2-targeted therapy to treat HER2-positive and hormone receptor (HR)-positive metastatic breast cancer in patients who cannot tolerate first-line chemotherapy. Moreover, data on the effectiveness and safety of cyclin-dependent kinase 4/6 inhibitors combined with trastuzumab and endocrine therapy as a first-line treatment for HER2-positive and HR-positive metastatic breast cancer are limited.

**Patient concerns:**

A 50-year-old premenopausal woman was with epigastric pain for more than 20 days. Ten years ago, she was diagnosed with left breast cancer and underwent surgical treatment, chemotherapy, and endocrine therapy.

**Diagnoses:**

After relevant examination, the patient was diagnosed with liver, lung, and left cervical lymph node metastatic HER2-positive and HR-positive carcinoma from the left breast after systemic therapy.

**Interventions:**

The laboratory investigations showed that the patient’s liver function was seriously damaged due to the liver metastases, and the patient was assessed as unable to tolerate chemotherapy. She was treated with trastuzumab, leuprorelin, letrozole, and piperacillin combined with percutaneous transhepatic cholangic drainage.

**Outcomes:**

The patient’s symptoms were relieved, her liver function returned to normal, and the tumor showed partial response. Neutropenia (Grade 3) and thrombocytopenia (Grade 2) occurred during treatment but improved after symptomatic treatment. To date, the progression-free survival of the patient is over 14 months.

**Lessons:**

We believe that trastuzumab, leuprorelin, letrozole, and palbociclib is a feasible and effective treatment for HER2-positive and HR-positive metastatic breast cancer in premenopausal patients who cannot tolerate first-line chemotherapy.

## 1. Introduction

Human epidermal growth factor receptor 2 (HER2)-positive and hormone receptor (HR)-positive breast cancer is a special type of breast cancer, representing an estimated 10% of all breast cancer subtypes.^[1]^ For patients with advanced or metastatic disease, chemotherapy plus HER2 targeted therapy is commonly recommended as a first-line treatment according to current international guidelines.^[2]^ However, some patients cannot tolerate the toxicity of chemotherapy because of their poor physical condition. Therefore, chemotherapy-free treatment is necessary. Here, we summarize a chemotherapy-free strategy for HER2-positive and HR-positive metastatic breast cancer patients as a first-line treatment. The patient described in this case showed significant improvement in survival following therapy.

## 2. Case report

### 2.1. Case presentation

A 50-year-old premenopausal woman was with epigastric pain for more than 20 days was admitted to our hospital on September 27, 2021. A space-occupying lesion (about 8 cm in size) in her liver was identified by computed tomography (CT) scan from another hospital before admission. In 2011, the patient was diagnosed with left breast cancer by the Thoracic and Breast Surgery department of our hospital and underwent a modified radical mastectomy. The pathological features and immunohistochemistry results of the left breast cancer were as follows: invasive ductal carcinoma; ER (40% +), partial response (PR) (40% +), HER2 (+++), ki-67 (50%+). After the operation, she was treated with 6 courses of fluorouracil, epirubicin, and cyclophosphamide and tamoxifen endocrine therapy for 5 years; no tumor recurrence or metastasis was found during the intermittent follow-up period. There was no history of tobacco or alcohol consumption, and no relevant family medical history.

### 2.2. Admission examination

**Figure 1.**
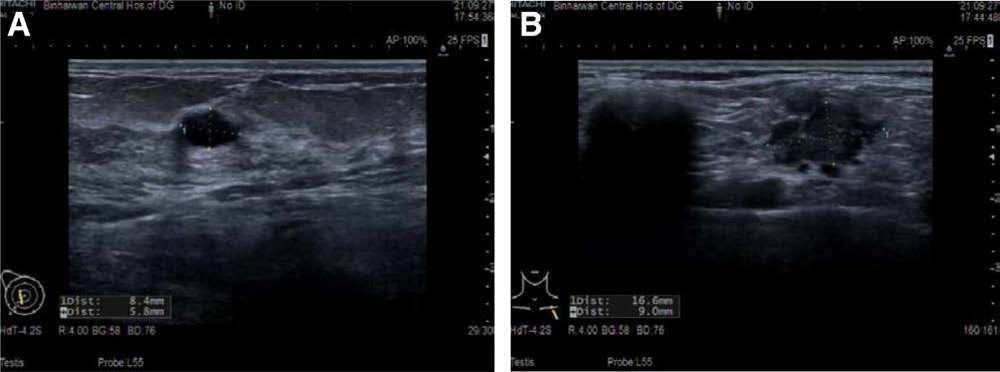
Color ultrasound of (A) a hypoechoic area in the right breast, about 8 mm × 6 mm in size, considering BI-RADS Class 4a; (B) enlarged lymph nodes in the left Cervical Region VII, approximately 17 mm × 9 mm in size.

**Figure 2.**
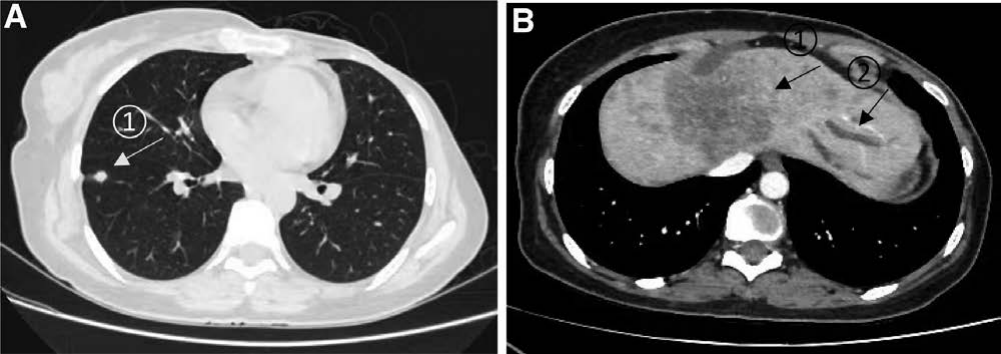
Computed tomography (CT) scan of (A) a small nodule in the lower lobe of the right lung, about 8 mm in diameter (arrow **①**), considered to be a metastatic tumor, (B) multiple low-density lesions in the left lobe of the liver with a maximum diameter of approximately 73 mm (arrow **①**), considered to be a metastatic tumor; obvious dilatation of the intrahepatic bile ducts was present in the left lobe of the liver(arrow **②**).

**Figure 3.**
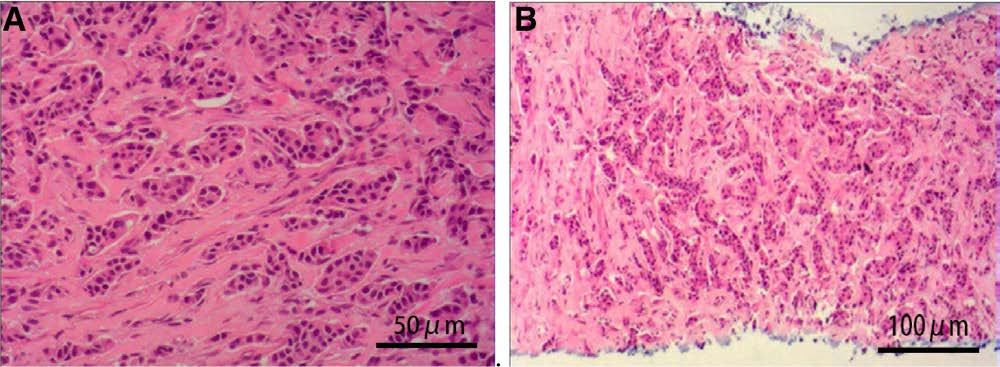
Pathological features. (A) enlarged lymph node in the left neck: adenocarcinoma, (B): liver lesions: adenocarcinoma; immunohistochemistry results: ER (~ 80% +), PR (~ 60% +), HER2 (+++), P63 (−), ki-67 (~60%+), P120 (+++), E-cadherin (+++), GATA-3 (+++), CK20 (−), EGFR (+). The combination of hematoxylin–eosin and immunohistochemistry results consistently suggested hepatic metastasis of invasive ductal carcinoma of the breast. HER2 = human epidermal growth factor receptor 2, PR = partial response.

Physical examinations revealed a 1 cm × 2 cm enlarged lymph node in the left neck and removal of the left breast. Liver function test results were as follows: alanine transaminase, 95.00 U/L (↑); aspartate transaminase, 99.00 U/L (↑); transglutaminase, 556.00 U/L (↑); alkaline phosphatase, 219.00 U/L (↑); total bilirubin, 144.90 umol/L (↑); and direct bilirubin, 96.79 umol/L (↑). Tumor marker tests were as follows: alpha-fetoprotein, normal; carcinoembryonic antigen, 7.53 ng/mL (↑); carbohydrate antigen 15 to 3, 300.0 u/mL (↑); carbohydrate antigen 125, 331.3 u/mL (↑); and carbohydrate antigen 19 to 9, 88.58 u/mL (↑). Other laboratory findings were unremarkable. Color ultrasound of the breast and armpit plus neck showed a hypoechoic area in the right breast, approximately 8 mm × 6 mm in size, considered as BI-RADS Class 4a. An enlarged lymph node, approximately 17 mm × 9 mm in size, was found in the left Cervical Region VII (Fig. 1). CT scan of the chest and abdomen revealed the following findings: A small nodule in the lower lobe of the right lung, approximately 8 mm in diameter, indicating a metastatic tumor; and Multiple low-density lesions in the left lobe of the liver with a maximum diameter of approximately 73 mm, obvious dilatation of the intrahepatic bile ducts of the left lobe of the liver, and no obvious dilatation in the common bile duct (Fig. 2). A whole-body bone scan showed no abnormalities. An ultrasound-guided biopsy of the liver and enlarged lymph nodes in the left neck revealed adenocarcinoma. Immunohistochemistry results were as follows: ER (80% +), PR (60% +), HER2 (+++), ki-67 (60%+), P63 (−), P120 (+++), E-cadherin (+++), GATA-3 (+++), CK20 (−), and EGFR (+). The results of hematoxylin–eosin and immunohistochemistry were consistent with hepatic metastasis of invasive ductal carcinoma of the breast (Fig. 3). The patient was diagnosed with liver, lung, and left cervical lymph node metastatic HER2-positive and HR-positive carcinoma from the left breast after systemic therapy.

### 2.3. Treatment

After admission, the patient was treated with liver-protective and bilirubin-lowering therapies. However, the patient’s liver function and bilirubin levels did not improve significantly, and we concluded that the patient could not tolerate chemotherapy. We treated her with trastuzumab (the initial loading dose was 8 mg/kg, subsequent dosing was 6 mg/kg every 3 weeks), leuprorelin (3.75 mg every 4 weeks), letrozole (2.5 mg every day), and piperacillin (125 mg, every day, 2 weeks on/1 week off schedule). The patient also underwent percutaneous transhepatic cholangic drainage.

### 2.4. Outcome and follow-up

**Figure 4.**
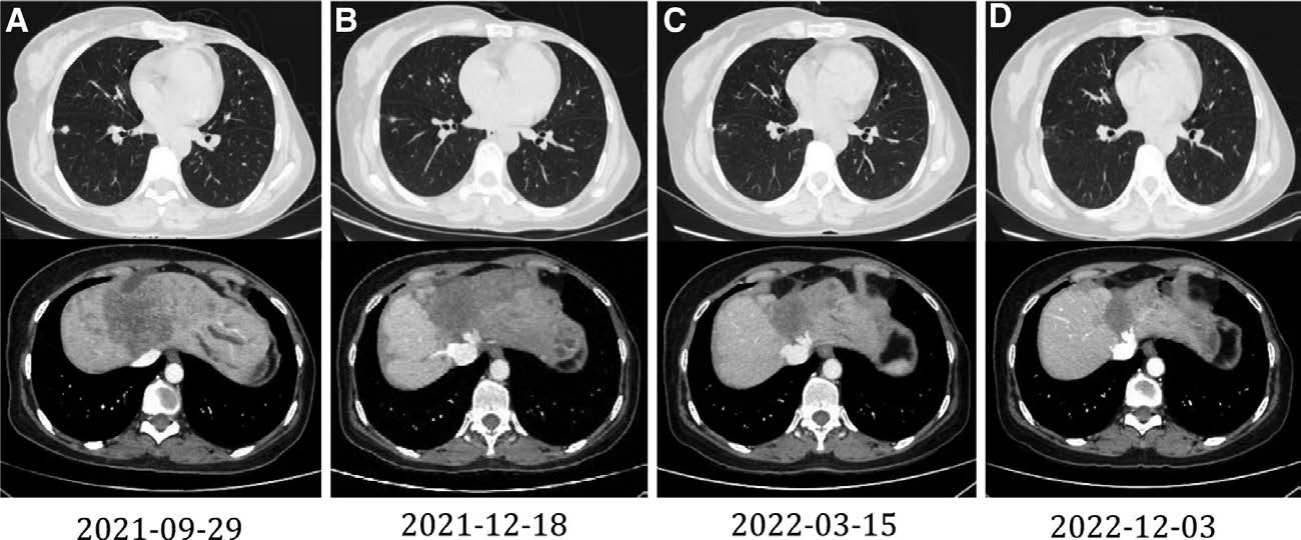
Changes in lung and liver lesions on CT scan. (A) Before treatment (2021-09-29), (B) After 2 months of treatment (2021-12-18), (C) After 5 months of treatment (2022-03-15), (D) After 14 months of treatment (2022-12-03). CT = computed tomography.

After 2 months of treatment, the patient’s liver function and bilirubin level improved and tumor markers decreased remarkably. In addition, the enlarged lymph node in the left neck disappeared. CT scan showed that the small nodule in the lower lobe of the right lung was smaller than that in the front. The low-density lesions in the left lobe of the liver were slightly larger than those in the front, with a maximum diameter of approximately 87 mm; however, the intrahepatic bile ducts were not dilated. The tumor response was evaluated as stable disease, as per the RECIST guideline (version 1.1). After 5 months of treatment, the patient’s liver function, bilirubin level, and tumor markers returned to normal. CT scan showed that the small nodule in the lower lobe of the right lung had almost disappeared, and the low-density lesions in the left lobe of the liver were reduced to a maximum diameter of approximately 66 mm. The tumor response was evaluated as stable disease. After 14 months of treatment, CT scan showed that the small nodule in the lower lobe of the right lung also basically disappeared, and the low-density lesions in the left lobe of the liver were significantly reduced to a maximum diameter of approximately 34 mm (Fig. 4). The tumor response was evaluated as a PR. During treatment, neutropenia (Grade 3) and thrombocytopenia (Grade 2) occurred, which improved after symptomatic treatment. To date, the progression-free survival (PFS) of the patient is over 14 months.

## 3. Discussion

At present, many clinical studies on chemotherapy-free strategies in HER2-positive and HR-positive advanced or metastatic breast cancer have been carried out, and some have achieved good results. The Tandem III study showed that trastuzumab plus anastrozole significantly increased PFS in patients with HER2-positive and HR-positive advanced breast cancer than anastrozole (4.8 months vs 2.4 months, *P* = .016) does, and improved overall survival and the overall response rate (ORR) to some extent.^[3]^ The PERTAINI Phase II study showed that in newly diagnosed postmenopausal women with HER2-positive and HR-positive advanced breast cancer, trastuzumab and pertuzumab plus AI or docetaxel significantly improved PFS (18.89 months vs 15.8 months, HR 0.65, 95% CI 0.48–0.89; *P* = .007) than trastuzumab and AI or docetaxel, demonstrating the superiority of HER2-dual-targeted therapy.^[4]^ The SYSUCC-002 Phase III study demonstrated that first-line endocrine plus trastuzumab therapy was not inferior to chemotherapy plus trastuzumab therapy for HER2-positive and HR-positive metastatic breast cancer in terms of PFS (19.2 months vs 14.8 months, *P* < .0001) and overall survival (33.9 months vs 32.5 months, *P* = .090).^[5]^

Cyclin-dependent kinases (CDK) are a family of molecules that play a key role in the control of cell division and represent an important therapeutic target. Selective CDK4/6 inhibitors have been approved for the treatment of patients with HER2-negative and HR-positive metastatic breast cancer; they are now the standard of care in first-line treatment and a valid option in second or further lines in combination with endocrine therapy.^[6,7]^ However, there is limited data on the effectiveness and safety of CDK4/6 inhibitors in HER2-positive and HR-positive metastatic breast cancer. Using immortalized breast cancer cell lines, Finn et al^[8]^ demonstrated that growth inhibition induced by palbociclib was greater in the luminal ER + subtype (including HER2-amplified cells) than in non-luminal ones. Moreover, palbociclib demonstrated a synergistic effect when combined with trastuzumab in HER2-positive cell lines.^[8]^ El Chaarani et al^[9]^ investigated the addition of palbociclib to a combination of trastuzumab and pertuzumab in HER2-positive breast cancer cell lines and confirmed that palbociclib was effective in both resistant and nonresistant HER2-positive cells. In the monarcHER phase II study, patients with HER2-positive and HR-positive advanced breast cancer who had received at least 2 prior HER2-target therapies were treated with trastuzumab plus fulvestrant and abecile. These patients showed a significantly increased ORR (33% vs 14%, odds ratio 3.2, 95% CI 1.4–7.1, *P* = .0042) and PFS (8.3 months vs 5.7 months; HR 0.67, 95% CI 0.45–1.00, *P* = .05) compared with the patients treated with trastuzumab and investigator-selected chemotherapy.^[10]^ The LORDSHIPS phase IB study showed that patients with HER2-positive and HR-positive recurrent or metastatic breast cancer who were treated with letrozole, pyrrotinib, and dalcyril had an overall ORR of 66.7% and a median PFS of 11.3 months. Among them, the ORR was 85.7% and the median PFS was not reached in the first-line treatment. The toxicity of this treatment was tolerable.^[11]^

In this case, we chose trastuzumab, leuprolide, letrozole, and piperacillin as first-line treatment regimens and achieved good therapeutic effects. The reasons for successful treatment in this case as follows: Expression of HR (ER 80% +, PR 60% +) and the biological characteristics of the patient’s tumor were better than those with low HR expression, closer to HER2-negative and HR-positive breast cancers. The patients enrolled in the SYSUCC-002 study also had tumors with higher HR expression (immunohistochemistry ≥ 10%) and good biological characteristics, and their tumor-free intervals were over 12 months. For patients with low HR expression (immunohistochemistry < 10%), the biological characteristics of the tumor are poor and close to those of HER2-positive and HR-negative breast cancer, which may not be suitable for the treatment of our case; Application of CDK4/6 inhibitor: CDK4/6 inhibitors are mainly used in the treatment of HER2-negative and HR-positive breast cancer, but there is currently limited evidence regarding their use in the treatment of HER2-positive and HR-positive breast cancer. The monarc HER and LORDSHIPS clinical studies have preliminarily explored the efficacies of CDK4/6 inhibitor and reported positively. We look forward to further large-scale phase III clinical studies needed to confirm these results.

## 4. Conclusion

In this case report, we confirmed that trastuzumab, leuprolide, letrozole, and piperacillin may be an effective treatment strategy for HER2-positive and HR-positive metastatic breast cancer in premenopausal patients who cannot tolerate chemotherapy as a first-line treatment. It can prolong the survival of patients with this disease and provide more treatment possibilities.

## Acknowledgements

We gratefully acknowledge the participation of our patients in this study.

## Author contributions

**Conceptualization:** Maoben Sun.

**Data curation:** Maoben Sun, Min Chen.

**Formal analysis:** Maoben Sun.

**Investigation:** Min Chen.

**Methodology:** Maoben Sun.

**Project administration:** Maoben Sun.

**Resources:** Maoben Sun.

**Writing – original draft:** Maoben Sun.

**Writing – review & editing:** Liangzhen Cai.
